# The Role of Voice Acoustics in Depression Assessment: Findings From Bibliometric Analysis, Literature Review, and Meta‐Analysis

**DOI:** 10.1155/da/5592230

**Published:** 2026-04-24

**Authors:** Lady Catherine Cantor-Cutiva, Andrés Carrillo-González, Ahmed M. Yousef, Mark Berardi, Eric J. Hunter

**Affiliations:** ^1^ Department of Audiology and Speech-Language Pathology, East Tennessee State University, Johnson City, Tennessee, USA, etsu.edu; ^2^ Department of Occupational Health and Safety, Corporación Universitaria Minuto de Dios – UNIMINUTO, Bogotá, Colombia, uniminuto.edu; ^3^ Center for Laryngeal Surgery and Voice Rehabilitation, Massachusetts General Hospital, Boston, Massachusetts, USA, harvard.edu; ^4^ Department of Surgery, Harvard Medical School, Boston, 02115, Massachusetts, USA, harvard.edu; ^5^ Department of Communication Sciences and Disorders, The University of Iowa, Iowa City, Iowa, USA, uiowa.edu

**Keywords:** depression, digital biomarkers, mental health, speech analysis, voice

## Abstract

**Background:**

Depression is a prevalent mental health disorder that significantly impairs psychosocial functioning and quality of life. Recent advances in health sciences and digital technologies have highlighted the potential of voice acoustic parameters as objective indicators of health status, including depression.

**Methods:**

A bibliometric analysis, systematic literature review, and meta‐analysis were conducted to consolidate and critically evaluate the current evidence regarding the relationship between voice acoustic parameters and depression. The search was performed in January 2024 across seven databases, including PubMed, Web of Science, and Scopus. Studies were included if they involved participants with clinically significant depression, identified either through formal diagnostic criteria or through validated depression rating scales with established clinical thresholds and explicitly reported voice acoustic parameters. A total of 31 potential publications were identified and analyzed, and after full‐text reading, 17 publications were included. Only six out of the 17 included studies reported sufficient numerical fundamental frequency (fo) data for meta‐analysis; other parameters could not be synthesized quantitatively due to a lack of extractable values.

**Results:**

The bibliometric analysis suggests an evolution from studies identifying “valid” assessment tools towards the modeling of potential discriminatory factors. The mean difference (MD) suggests a decreased fo of 1.82 Hz among participants identified with depression compared to participants identified as the control group. However, the difference between the groups was not statistically significant (*Z* test = 0.58; *p*‐value 0.56).

**Conclusions:**

Voice acoustic parameters seem to have the potential to be noninvasive, cost‐effective biomarkers for measuring and monitoring depression symptomatology. Although there was a trend of decreased fo of 1.82 Hz among participants identified with depression compared to participants identified as the control group, the meta‐analysis suggests a nonsignificant difference in average values.

## 1. Introduction

Clinical depression is a common mental health disorder that markedly impairs psychosocial functioning and substantially reduces overall quality of life [1]. It is among the most prevalent and debilitating psychiatric conditions worldwide, affecting an estimated 280 million people and ranking as a leading cause of disability [2]. Between 1990 and 2017, the global number of depression cases increased from 172 to 258 million, representing a 49.86% rise [3]. By 2030, depression is projected to become the leading cause of global disease burden [4]. This burden is further compounded by the fact that only about one‐third of individuals with depression receive treatment. This disparity is most pronounced in low‐income countries, as classified by the World Bank (16.8%), compared to high‐income countries (48.3%) [5].

The etiology of depressive disorders is widely recognized as multifactorial, involving a complex interplay of genetic predispositions, environmental influences, and lifestyle‐related factors [6, 7]. Nevertheless, depression diagnosis involves multiple methodological approaches, including structured clinical interviews, standardized assessment tools, longitudinal observation, and collateral information gathering. While these methods follow established protocols, they still incorporate subjective elements from both patient reporting and clinician judgment, which can introduce reliability concerns in both diagnosis and symptom monitoring [8]. Self‐reporting, for example, depends heavily on a patient’s willingness, ability, and honesty in communicating symptoms, moods, and cognitions, even though the disorder inherently compromises motivation and outlook [9].

Over the past 15 years, advances in health sciences and digital technologies have underscored the potential of voice acoustic parameters as objective indicators of health status [10], including cognitive function [11], mood status, neurological, cardiorespiratory, and pediatric conditions [12]. These parameters have emerged as complementary, less invasive, and easier to repeat biomarkers, such as blood, genetic markers, and fMRI, that have struggled to achieve clinical significance [13]. Depression, beyond its well‐known cognitive and behavioral manifestations, also presents subtle yet measurable alterations in voice acoustic features. For example, research has shown that individuals with depression often exhibit lower fundamental frequency (fo), which may reflect hypokinetic disturbances; elevated jitter and shimmer (measures of frequency and amplitude variability, respectively), which correlate with depression severity; and reduced spectral features such as spectral tilt and glottal‐to‐noise excitation (GNE) ratio, which can provide significant discriminatory power [14]. Likewise, Mel‐frequency cepstral coefficients (MFCCs), particularly MFCC 2 (the second dimension), differ markedly between depressed and nondepressed individuals, indicating an energy contrast in the 2000–3000 Hz frequency range [15]. Decreased vocal loudness and energy further characterize the speech of depressed individuals, potentially reflecting their affective state [16].

Investigating voice acoustic parameters as potential biomarkers for depression has several advantages. First, voice analysis is noninvasive and cost‐effective, requiring minimal equipment [17, 18]. Second, it allows for continuous monitoring of disease progression and treatment efficacy, essential for long‐term management [19]. Third, remote voice analysis improves healthcare access for populations in geographically isolated or underserved regions, which is crucial for ongoing monitoring [18, 20]. As a digital biomarker, voice analysis may bridge the gap between subjective clinical observation and objective physiological metrics, providing insight into how depressive symptoms manifest and evolve.

Several systematic reviews have investigated the connection between voice acoustics and depression. Notably, the review by Cummins et al. [9] is the most relevant to the objectives listed above, as it focused on identifying speech characteristics and voice acoustic parameters as potential biomarkers for depression and suicidality. However, this review was conducted nearly a decade ago, and advancements in technology and data availability make it necessary to conduct an updated analysis of the state of the field. More recent studies have also used deep learning and machine learning applications using speech parameters as input to detect depression; those using such techniques are beyond the scope of our review [21, 22]. Furthermore, given the breadth of speech measures in previous reviews, our systematic review specifically focuses on voice acoustic parameters that reflect laryngeal and aerodynamic function. While we did not exclude studies that also measured other acoustic or linguistic features, such as suprasegmental or temporal features, our primary interest lies in parameters reflecting physiological aspects of voice production. This targeted approach examines physiological changes in vocal fold vibration and respiratory control that may be directly affected by depression’s psychomotor symptoms, distinct from cognitive–linguistic processes captured by other speech measures [23]. By isolating these parameters, we aim to identify biomarkers more directly linked to the physiological manifestations of depression and less influenced by semantic content or cognitive factors.

The study of voice acoustic parameters in depression involves a multidisciplinary approach from speech–language pathology and psychology, along with other disciplines like linguistics and psychiatry, to the assessment of depression, illustrating the value of interdisciplinary mental health research. To better understand the physiology of voice and speech, speech–language pathologists and speech scientists analyze acoustic patterns from voice and speech. Psychologists contribute a deep understanding of depression’s manifestation, assessment protocols, and treatment approaches. This collaboration enables a more comprehensive approach to understanding how depression affects voice production and how these changes might be leveraged for clinical applications.

Given this context, the present systematic literature review aims to consolidate and critically evaluate the current evidence base regarding the relationship between voice acoustic parameters and depression. This review is particularly timely given the rapid advancement of technology‐enabled mental health assessment tools and the growing need for objective, accessible methods of depression screening and monitoring. By integrating perspectives from both speech–language pathology and psychology, this review will provide a comprehensive foundation for future research and clinical applications in this emerging field.

## 2. Methods

### 2.1. Literature Search

The search was performed in January 2024. In the first stage (screening), indexed publications focused on studying the relationship between acoustic voice measurements and any health condition that affects voice production were considered. A wide range of acoustic voice metrics was targeted. This broad inclusion criterion ensured that both primary voice disorders and conditions where voice changes are secondary symptoms were detected.

**Figure 1:**
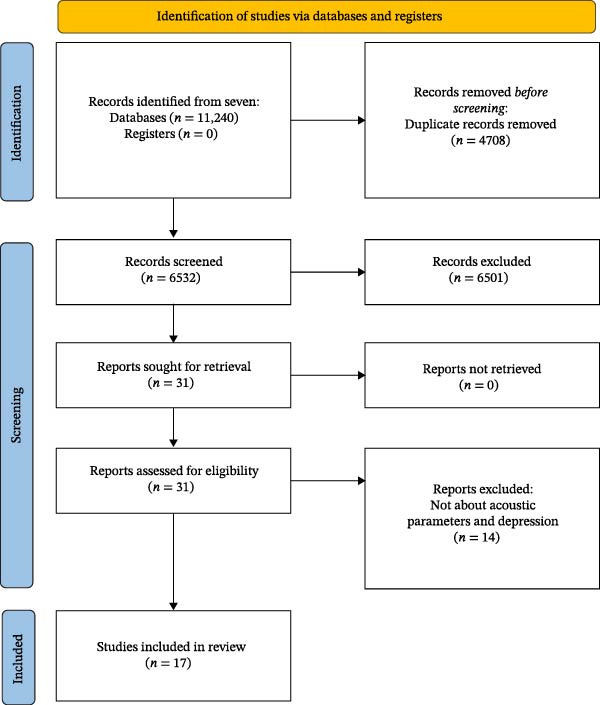
PRISMA 2020 flow diagram for systematic review on the relationship between voice acoustic parameters and depression.

Two Medical Subject Headings (MeSH) terms were identified in the search string: “voice disorders” and “speech acoustics” to cover a wide range of acoustic measurements and voice pathology during the literature review. Seven databases were used to identify potential manuscripts on this topic. The databases included PubMed, Web of Science, Scopus, EBSCO (Academic Search Elite), Science Direct, BVS, and Scielo. The BVS and Scielo databases offer a broad search scope, including Spanish and Portuguese publications. No restrictions on the type of publication were set during the screening phase. A total of 11,240 publications were identified: 2096 from PubMed, 2290 from Web of Science, 2874 from Scopus, 865 from EBSCO, 1960 from Science Direct, and 1155 from both BVS and Scielo. Since having duplicate titles was likely because different databases were used, the merging of duplicates was performed using Zotero (a digital citation manager, https://www.zotero.org/) and Rayyan (a systematic review tool, https://www.rayyan.ai/). After removing duplicates, the total number of collected publications to be screened was 6532. As the final step, we identified 31 potential publications on voice acoustic parameters and depression (Figure 1). This study was registered in the International Prospective Register of Systematic Reviews (PROSPERO) (CRD42024623455).

### 2.2. Publication Selection

Studies were included if they satisfied all the following initial selection criteria: (1) they were written either in English, Spanish, or Portuguese; (2) they involved participants with clinically significant depression, identified either through formal diagnostic criteria (e.g., Diagnostic and Statistical Manual of Mental Disorders [DSM]) or through validated depression rating scales with established clinical thresholds; and (3) they explicitly reported voice acoustic parameters. Ten variables were then extracted from each publication: language, year, journal, country, continent, sample size, depression assessment, control group subjects, acoustic parameters, and main results.

### 2.3. Bibliometric Analysis and Keyword Analysis

The bibliometric analysis was conducted to systematically examine the publication trends. Co‐occurrence networks were plotted to cluster frequently occurring terms identified across the selected papers, facilitating the detection of thematic patterns and relationships within the reviewed literature. VOSviewer (https://www.vosviewer.com/) was used to perform the co‐occurrence analysis to identify the relationship between the terms in the manuscripts. Co‐occurrence network analysis was also used to determine citation occurrence within terms related to voice acoustic parameters and depression.

### 2.4. Quality Assessment of the Included Publications

We employed the Quality Assessment Tool for Quantitative Studies [24], which evaluates eight key components on a scale of 1–3, where a score of one signifies strong quality, two indicates moderate quality, and three corresponds to weak quality. The tool examines selection bias, study design, confounders, blinding, data collection methods, withdrawals and dropouts, intervention integrity, and analyses. Each publication’s overall rating was then determined by counting how many components received a weak score: studies with no weak scores were classified as strong, those with one weak score were classified as moderate, and those with two or more weak scores were classified as weak. Two authors independently assessed the methodological quality of every included publication.

### 2.5. Meta‐Analysis of Included Publications

To determine the relationship between voice acoustic parameters and depression, a random‐effects meta‐analysis was conducted. The random‐effects method was employed to account for the variation in population parameters across studies, and the weights used to calculate the summary estimate were adjusted accordingly [25]. The mean difference (MD) was chosen as the effect measure for the meta‐analysis. Heterogeneity was assessed using the *χ* ^2^ test and the *I* ^2^ test, where the *I* ^2^ statistic represents the percentage of variation across studies that can be attributed to heterogeneity rather than chance. The interpretation of the observed *I* ^2^ value depends on the effect sizes, the strength of evidence for heterogeneity, and the direction of effects [26]. The meta‐analysis was performed using Review Manager software—RevMan (https://revman.cochrane.org). Given the limited number of included studies, the exploration of possible causes of heterogeneity through methods such as subgroup analysis or meta‐regression will not be deemed necessary for this meta‐analysis.

### 2.6. Publication Bias

Publication bias in the meta‐analysis was assessed by examining funnel plots. Funnel plots are graphical representations that help evaluate potential publication bias by assessing the symmetry of the distribution of study results [27]. Asymmetry in the funnel plot can indicate the presence of publication bias, suggesting that studies with smaller sample sizes or nonsignificant results may be less likely to be published [28]. By including funnel plot analysis, we aimed to consider and address the potential impact of publication bias on the overall findings of the meta‐analysis.

## 3. Results

### 3.1. Characteristics of the Publications (Bibliometric Analysis)

**Figure 2:**
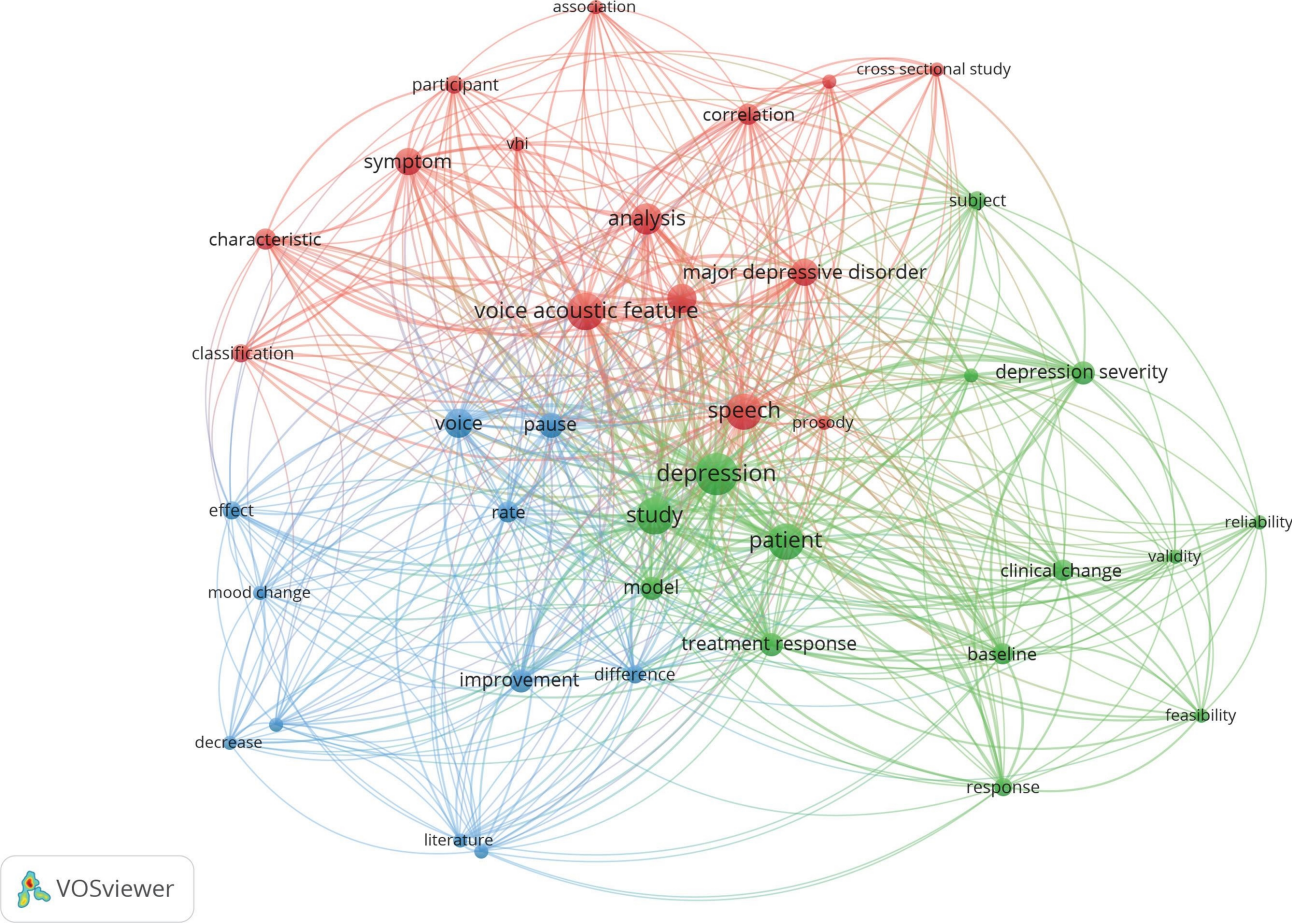
Co‐occurrence network of terms extracted from titles and abstracts.

**Figure 3:**
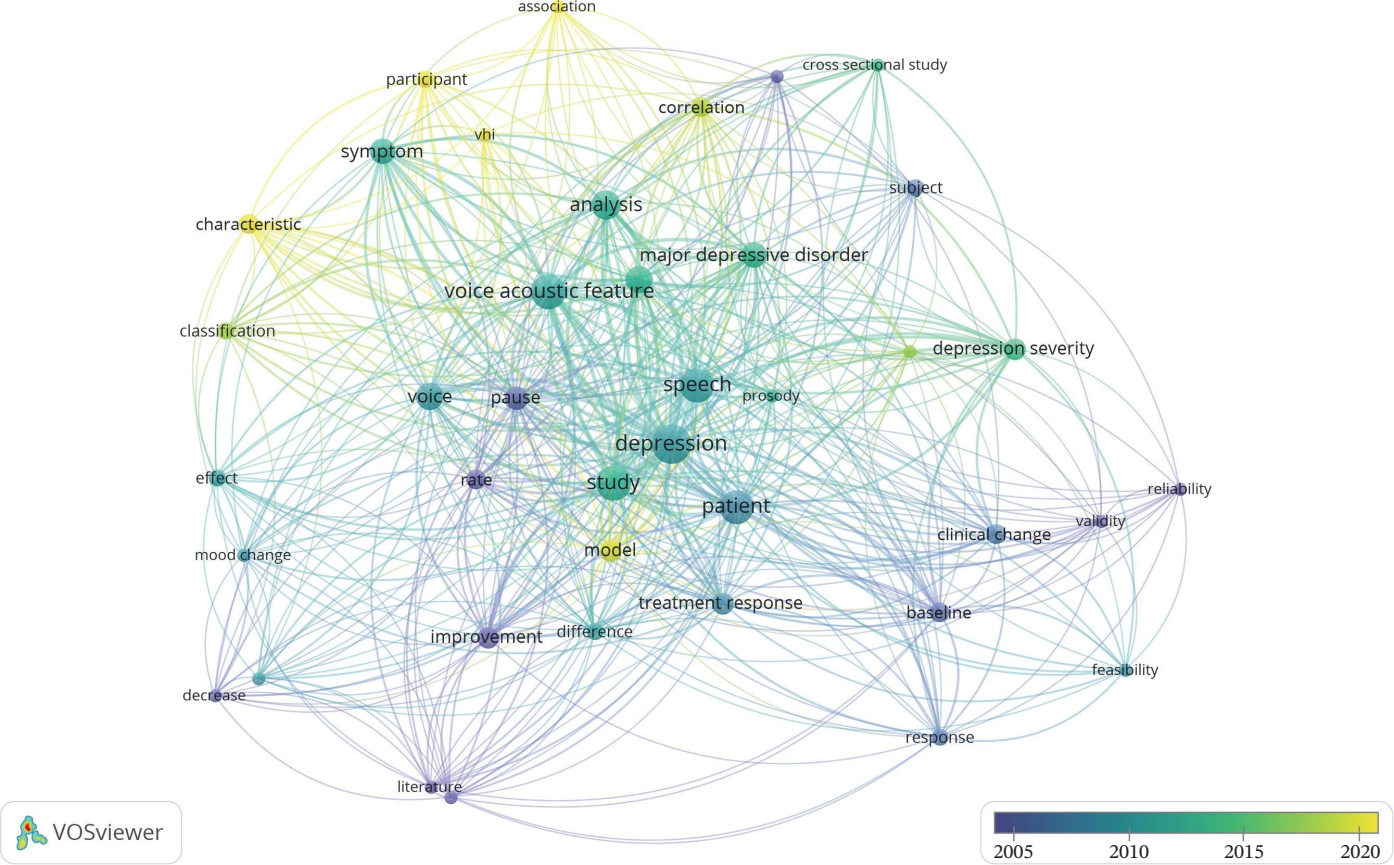
Overlay by average publication per year visualization of the co‐occurrence network of terms extracted from titles and abstracts.

**Figure 4:**
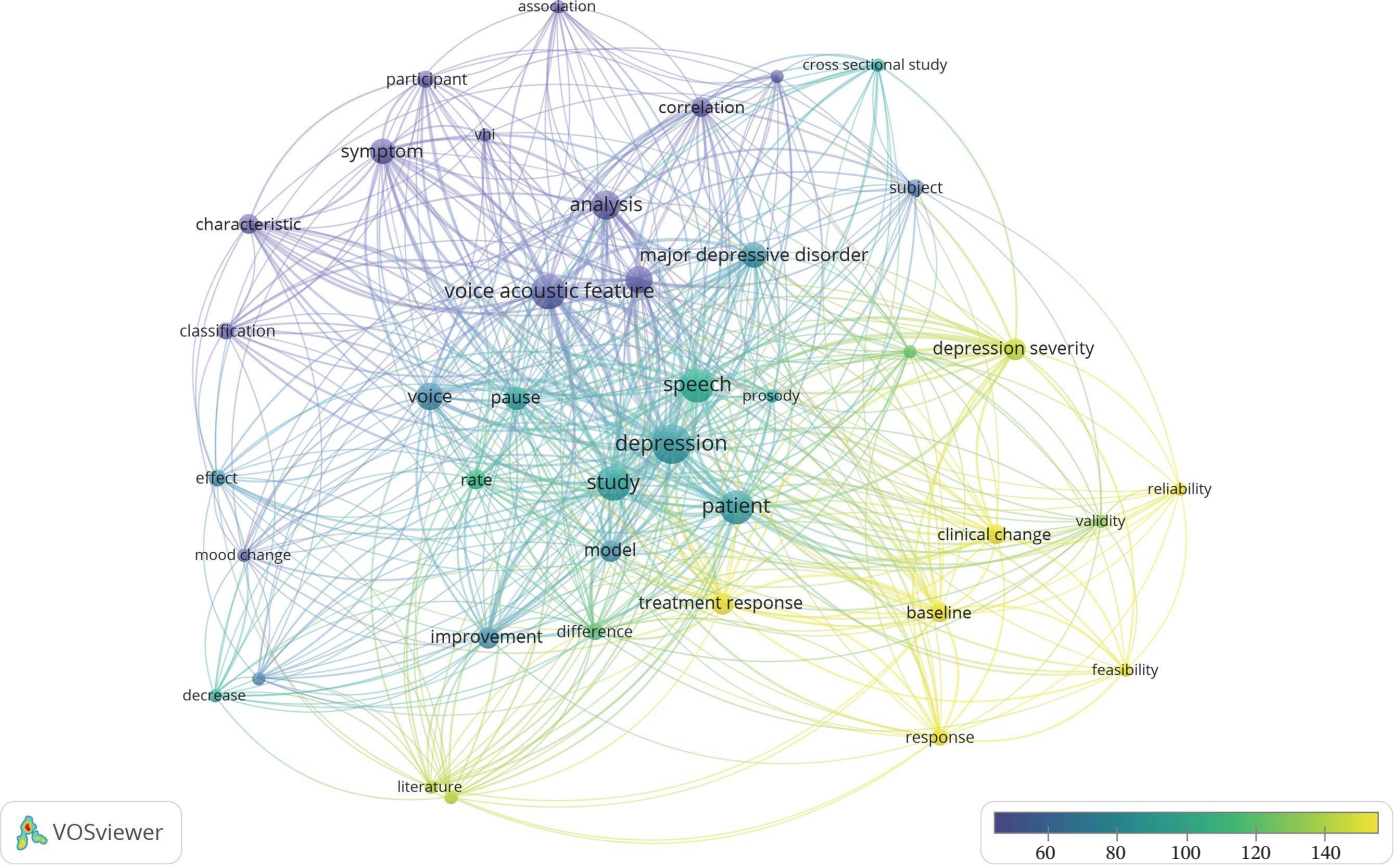
Overlay by average citations visualization of the co‐occurrence network of terms extracted from titles and abstracts.

After the full‐text review, 17 manuscripts were included in this study. Figures 2–4 present the term co‐occurrence networks generated through VOSviewer as part of the bibliometric analysis. These maps summarize how key concepts have been used across the body of literature on depression and voice acoustics, helping to identify thematic clusters and methodological patterns that guided the subsequent qualitative review. Figure 2 shows three major clusters centered around symptom severity, treatment response, and psychometric evaluation, indicating the primary conceptual domains represented in the field. Figure 3 illustrates the temporal evolution of terminology, with earlier studies focusing on psychometric validation (“validity” and “reliability”) and more recent publications emphasizing modeling approaches (“association” and “model”).

Figure 4 displays citation‐weighted co‐occurrence patterns, highlighting terms associated with the most influential studies (“depression severity” and “clinical change”) and underscoring the areas with limited empirical attention (“VHI” and “classification”). Together, these visualizations contextualize the current evidence base and support the rationale for narrowing the literature review and meta‐analysis.

Table 1 provides an integrative overview of the studies included in the bibliometric analysis and literature review, listing the first author, country, publication year, sample size, definition and assessment methods of depression, and voice acoustic parameters. There was considerable diversity in the sample sizes of the included studies, ranging from smaller cohorts of 7−16 participants to larger groups exceeding 150 participants. Regarding the definition of depression, the methodological approach consistently demonstrated an integration of clinical judgment, with quantitative measurement tools. Researchers typically employed a comprehensive strategy that combined standardized diagnostic manual criteria, validated rating scales, and clinical psychiatric evaluation.

**Table 1:** Overview of the included studies listing the first author, country, publication year, sample size, definition and assessment methods of depression, and voice acoustic parameters.

First author last name, country, publication year	Sample size		Definition of depression	Assessment methods for depression	Voice acoustic parameters
	Cases	Controls			
Songur et al. [29], Turkey, 2022	38 adults with rheumatoid arthritis (RA)	42 healthy adult volunteers	The paper defines depression based on the DSM‐V criteria, the mini‐international neuropsychiatric interview (MINI), the Hamilton depression rating scale (HAM‐D), and self‐rating depression scale (SDS)	Beck depression inventory (BDI) was used to determine the levels of the participants in terms of symptoms of depression	MPT, mean fo, jitter local, jitter abs, jitter rap, jitter ppq, shimmer local, shimmer dB, shimmer apq3, shimmer apq5, shimmer apq11, and NHR
Lu et al. [30], China, 2023	47 patients with functional dysphonia (FD)	22 controls	Depression is defined using the Hamilton anxiety and depression rating scale	The Hamilton anxiety and depression scales were used for psychological evaluation	Fundamental frequency, jitter, shimmer, noise‐to‐harmonic ratio, maximum phonation time
Wang et al. [31], China, 2023	47 college students	There was no control group included in the study	The paper discusses depression as a condition identified through the Hamilton depression rating scale (HAM‐D) and the patient health questionnaire‐9 (PHQ‐9)	Depression was identified using the self‐rated patient health questionnaire‐9 (PHQ‐9). Participants who met the criteria were further evaluated through a standardized HAMD‐17 telephone interview. A score of seven or higher on the Hamilton depression (HAM‐D) scale indicated the presence of depressive symptoms	fo, VUV, NAQ, QOQ, H1H2, PSP, MDQ, peak slope, Rd, Rd‐conf, creak, MCEP, HMPDM, HMPDD, peak‐to‐RMS, formant, MFCC‐deltas, and MFCC‐delta‐deltas
Flint et al. [32], Canada, 1992	30 patients with major depression (MD) + 30 patients with PD	31 normal controls	The article defines major depression based on the DSM‐III‐R criteria and a Montgomery‐Asberg depression rating Scale (MADRS) score of 15 or higher	Depression was defined based on the DSM‐III‐R criteria and a score of 15 or higher on the Montgomery‐Asberg depression rating scale (MADRS)	Mean fundamental frequency, fundamental frequency standard deviation, frequency change across a pitch glide measure, second formant transition, rate, vowel segment duration, speech pause time expressed as absolute time, percentage of the utterance, voice onset time, spirantization, and voice intrusion errors
Nilsonne [33], Sweden, 1987	16 depressed inpatients	There was no control group included in the study	The paper defines depression based on two sets of criteria: the Spitzer‐Endicott research diagnostic criteria (RDC) and the DSM‐III criteria	Depression was defined according to the Spitzer‐Endicott research diagnostic and DSM‐III criteria	Reading time, speech time, pause time, percent of reading time, mean fundamental frequency, standard deviation, rate of change of fundamental frequency and its standard deviation, and average speed of fundamental frequency change
Cummins et al. [34], Australia, 2015	150 from the audio/visual emotion challenge and workshop (AVEC) + 35 from the James Mundt 35‐speaker database (Mundt‐35)	Not reported	The article defines depression in the context of its operational framework. It utilizes two commonly used depression datasets: AVEC‐2013 dataset (using the Beck depression Inventory) and the Mundt‐35 dataset (using the quick inventory of depressive symptomatology)	Assessment was performed using the Beck depression inventory (BDI) and the quick inventory of depressive symptomatology (QIDS)	Mel‐frequency cepstral coefficients, average weighted variance, acoustic movement, and acoustic volume
Albuquerque et al. [35], Portugal, 2021	112 adult Portuguese speakers	There was no control group included in the study	The paper uses the hospital anxiety and depression scale (HADS) to assess depressive symptoms, focusing specifically on nonsevere cases	Anxiety and depression symptoms were assessed using the hospital anxiety depression scale (HADS), a self‐report questionnaire	Vowel fo, vowel formant frequencies, vowel duration, total speech duration, total pause duration, total recording duration, percent pause time, speech pause ratio, number of pauses, mean pause duration, mean speech duration, pause variability, speech variability, number of syllables, speech rate, speaking fo, and HNR
Kim et al. [36], Republic of Korea, 2023	153 patients with major depressive disorder (MDD)	165 healthy controls	The paper defines depression using standard clinical criteria. Specifically DSM criteria and through the Hamilton depression rating scale (HAM‐D) and the patient health questionnaire‐9 (PHQ‐9)	Board‐certified psychiatrists evaluated all patients according to the diagnostic and statistical manual of mental disorder criteria. The severity of depressive symptoms was assessed using the Hamilton depression rating scale (HAM‐D) and patient health questionnaire‐9 (PHQ‐9)	MFCCs, COMPARE, and eGeMAPS
Ooi et al. [37], Australia, 2014	The at‐risk (AR) group consisted of 15 adolescents	The not‐at‐risk (NAR) group consisted of 15 healthy controls	The paper defines depression based on the DSM‐IV diagnostic criteria	Depression was defined following the DSM‐IV depression screening criteria	Glottal timing, glottal frequency, jitter, shimmer, fundamental frequency, log energy, and TEO features derived from glottal waveform
Talavera et al. [38], Spain, 1994	20 depressive patients	20 normal subjects	The paper defines depression using multiple standardized criteria and measures, including the DSM‐III‐R criteria, the Hamilton depression scale (HAM‐D), the Zung self‐rating depression scale, and the Spielberger trait anxiety inventory (STAI)	Depression was assessed using Hamilton’s depression scale, the Zung’s self‐rating depression scale, and STAI Spielberger’s trail anxiety scale	Fundamental frequency (fo) in each time interval of analysis, fo standard deviation, 5% and 95% percentiles of the fo distribution, mean and SD of jitter‐small variations, pause and the phonation times, amplitude of acoustic waves, average value of all amplitudes within each utterance, vocal shimmer, and spectral energy distribution in four frequency bands
Alpert et al. [39], USA, 2001	12 participants with MDD	19 normal subjects	The article defines depression based on the DSM‐III‐R criteria and the Hamilton depression rating scale (HAM‐D)	Depression was defined using the DMS‐III‐R criteria for major depressive disorder and had a score of at least 18 on the 24‐item Hamilton depression rating scale	Average utterance duration, internal pause duration, average switching pause duration, percent of time talking, loudness, variance of voice level across all peaks, fundamental frequency (fo), and variance in fo
Berardi et al. [40], Germany, 2023	20 patients suffering from DSM‐IV schizophrenia spectrum disorder (SSD) + 20 from MDD	20 healthy controls	The article defines depression based on the DSM‐IV criteria and the Hamilton depression rating scale (HAM‐D)	Depression was assessed using the Hamilton depression scale, the scale for the assessment of negative symptoms, the scale for the assessment of positive symptoms, and the GAF global assessment of functioning	Speech rate, articulation rate, talking rate, pause duration, pause duration standard deviation, pause rate, fo SD, kurtosis, skewness, intensity SD, kurtosis, skewness, energy velocity, mean MFFC for coefficients one through 13, pauses per minute, three vocal‐tract‐variable‐based articulation coordination features, mean cepstral peak prominence smoothed (CPPS), CPPs SD, kurtosis, and skewness, low‐to‐high ratio mean (LHR), LHR SD, kurtosis, and skewness
Seifpanahi et al. [41], Iran, 2023	30 women with major depression	30 normal women	The paper defines depression using the Hamilton rating scale for depression (HRS‐D)	The depression severity was assessed using the Hamilton rating scale for depression (HRS‐D)	Means of switching pauses or latency, speaking rate, mean and standard deviation of fo, jitter, shimmer, HNR, and CPP
Mundt et al. [42], USA, 2012	165 participants	There was no control group included in the study	The article defines depression operationally using the DSM‐IV criteria, the Hamilton depression rating scale (HAM‐D) and the quick inventory of depressive symptomatology (QIDS‐C)	Depression was defined using the 17‐item clinician‐administered HAM‐D. Primary diagnosis of major depressive disorder based on DSM‐IV criteria. Clinical assessments of depression severity included clinician‐rated QIDS (QIDS‐C) and HAM‐D and a paper self‐report form of the QIDS (QIDS‐SR)	Total recording time, total vocalization time, total pause time, number of pauses, mean pause length, pause variability, percent pause time, speech/pause ratio, speaking rate, syllables/second, mean, SD, COV of fo, mean, SD, COV of F1, mean, SD, and COV of F2
Ellgring and Scherer [43], Switzerland, 1996	16 hospitalized patients	There was no control group included in the study	Depression was defined using the ICD‐9 diagnostic criteria	Depression was assessed by the clinical criteria of two psychiatrists	Speech rate, mean pause duration, number of pauses, mean fo, range of fo, and minimum fo
Mundt et al. [44], USA, 2007	35 patients	There was no control group included in the study	The paper defines depression using the Hamilton depression rating scale (HAM‐D) and the quick inventory of depressive symptomatologys(QIDS)	Patients completed a HAM‐D and quick inventory of depressive symptomatology (QIDS)	Pitch variability about fo, F1, and F2 was measured by the coefficient of variation, total recording duration, vocalization time, pauses while speaking, percent time pausing, vocalization/pause ratio, speaking rate, mean and variability of syllable durations, mean and variability of vocal intensities, and syllable rate
Cannizzaro et al. [45], USA, 2004	Seven participants	There was no control group included in the study	The article defines depression using the Hamilton depression rating scale (HDRS)	All participants were assessed using the HDRS 21 question rating scale for depression	Speaking rate, percent pause time, and pitch variation

Table 2 provides a summary of the included studies reporting depression scores and selected voice acoustic parameters results. As shown in the table, a wide array of voice acoustic parameters were reported. Seven of the 17 included studies (41%) reported at least one measure of fo, including mean fo and fo variability. Jitter, shimmer, and harmonics‐to‐noise ratio (HNR) were examined in two studies (12%). Noise‐to‐harmonics ratio (NHR) and maximum phonation time (MPT) were reported in one study (6%).

**Table 2:** Overview of the included studies reporting depression scores and selected voice acoustic parameters results.

First author last name, country, publication year	Depression scores		Fundamental frequency (Hz)		fo SD		Jitter local		Jitter abs		Shimmer local		Shimmer dB		NHR		HNR		MFCCs		MPT	
	Cases	Controls	Cases	Controls	Cases	Controls	Cases	Controls	Cases	Controls	Cases	Controls	Cases	Controls	Cases	Controls	Cases	Controls	Cases	Controls	Cases	Controls
Nilsonne [33], Sweden, 1987	CPRS: 2.1	NR	150.0	NR	15.0	NR	NR	NR	NR	NR	NR	NR	NR	NR	NR	NR	NR	NR	NR	NR	NR	NR
Flint et al. [32], Canada, 1992	MADRS: 33.60	MADRS: 0.39	F: 76.4 M: 61.8	F: 80.1 M: 62.0	F: 28.3 M: 22.5	F: 26.9 M: 27.1	NR	NR	NR	NR	NR	NR	NR	NR	NR	NR	NR	NR	NR	NR	NR	NR
Talavera et al. [38], Spain, 1994	HAM‐D: 25	HAM‐D: 1.7	NR	NR	NR	NR	NR	NR	NR	NR	NR	NR	NR	NR	NR	NR	NR	NR	NR	NR	NR	NR
Ellgring and Scherer [43], Switzerland, 1996	VAS: 75	NR	NR	NR	NR	NR	NR	NR	NR	NR	NR	NR	NR	NR	NR	NR	NR	NR	NR	NR	NR	NR
Alpert et al. [39], USA, 2001	HDRS: 24.8	NR	142.0	150.6	27.2	31.4	NR	NR	NR	NR	NR	NR	NR	NR	NR	NR	NR	NR	NR	NR	NR	NR
Cannizzaro et al. [45], USA, 2004	HDRS: 23.57	NR	NR	NR	NR	NR	NR	NR	NR	NR	NR	NR	NR	NR	NR	NR	NR	NR	NR	NR	NR	NR
Mundt et al. [44], USA, 2007	HAM‐D	NR	157.3	151.8	33.6	36.5	NR	NR	NR	NR	NR	NR	NR	NR	NR	NR	NR	NR	NR	NR	NR	NR
Mundt et al. [42], USA, 2012	HAM‐D: 22	NR	NR	NR	NR	NR	NR	NR	NR	NR	NR	NR	NR	NR	NR	NR	NR	NR	NR	NR	NR	NR
Ooi et al. [37], Australia, 2014	DSM‐IV	NR	NR	NR	NR	NR	NR	NR	NR	NR	NR	NR	NR	NR	NR	NR	NR	NR	NR	NR	NR	NR
Cummins et al. [34], Australia, 2015	BDI: >20	NR	NR	NR	NR	NR	NR	NR	NR	NR	NR	NR	NR	NR	NR	NR	NR	NR	NR	NR	NR	NR
Albuquerque et al. [35], Portugal, 2021	HADS_ D: >7	NR	F: 181.8 M: 151.5	F: 191.6 M: 124.9	F: 19.1 M: 24.9	F: 20.8 M: 20.7	NR	NR	NR	NR	NR	NR	NR	NR	NR	NR	F: 14.0 M: 12.2	F: 14.4 M: 10.3	NR	NR	NR	NR
Songur et al. [29], Turkey, 2022	RA and BDI: >15.8%	RA and BDI: 2.4%	F: 213.5 M: 138.9	F: 224.8 M: 141.1	NR	NR	F: 0.25% M: 0.31%	F: 0.23% M: 0.23%	F: 11.91 ms M: 23.99 ms	F: 10.49 ms M: 16.80 ms	1.91%	1.78%	NR	NR	0.0036	0.0040	NR	NR	NR	NR	F: 13.3 M: 11.6	F: 16.7 M: 18.7
Berardi et al. [40], Germany, 2023	HAM‐D: 6.15	HAM‐D: 0.82	NR	NR	NR	NR	NR	NR	NR	NR	NR	NR	NR	NR	NR	NR	NR	NR	NR	NR	NR	NR
Kim et al. [36], Republic of Korea, 2023	HAM‐D: 22.18 ‐‐ PHQ‐9 : 2.14	HAM‐D: 2.07 ‐‐ PHQ‐9 : 16.92	NR	NR	NR	NR	NR	NR	NR	NR	NR	NR	NR	NR	NR	NR	NR	NR	NR	NR	NR	NR
Lu et al. [30], China, 2023	HAM‐D: 25	NR	NR	NR	NR	NR	NR	NR	NR	NR	NR	NR	NR	NR	NR	NR	NR	NR	NR	NR	NR	NR
Seifpanahi et al. [41], Iran, 2023	HRS‐D: >15	NR	215.48	205.78	9.34	6.75	0.46	0.44	NR	NR	3.74	4.23	NR	NR	NR	NR	21.21	19.74	NR	NR	NR	NR
Wang et al. [31], China, 2023	HAM‐D: >17	NR	NR	NR	NR	NR	NR	NR	NR	NR	NR	NR	NR	NR	NR	NR	NR	NR	NR	NR	NR	NR

*Note:* fo SD, standard deviation of fundamental frequency; HAM‐D, Hamilton rating scale for depression.

Abbreviations: BDI, beck depression inventory; CPRS, comprehensive psychopathological rating scale; HADS, hospital anxiety and depression scale; HDRS, Hamilton depression rating scale; HNR, harmonics‐to‐noise ratio; MADRS, Montgomery‐Asberg depression rating scale; MFCCs, Mel‐frequency cepstral coefficients; MPT, maximum phonation time; NHR, noise‐to‐harmonics ratio; NR, not reported, PHQ‐9, patient health questionnaire‐9; VAS, visual analog scale.

More sophisticated acoustic measures, such as MFCCs, appeared in recent studies, indicating an evolution toward more complex acoustic analysis methods. Several studies also incorporated measures of voice quality, such as the HNR and cepstral peak prominence (CPP), providing additional dimensions for voice characterization.

Regarding depression symptomatology assessment methods, the Hamilton Depression Rating Scale (HAM‐D) stands out as the most commonly used quantitative assessment tool, utilized in 11 of the 17 studies. Several studies implemented multiple assessment tools, such as combining the Beck depression inventory (BDI) and Patient Health Questionnaire‐9 (PHQ‐9) with HAM‐D or utilizing the quick inventory of depressive symptomatology (QIDS) alongside other measures.

Table 3 shows that diverse speech and voice tasks to elicit samples for acoustic analysis were employed. Interestingly, all the included publications used text reading as part of their assessment protocol. Sustained phonation tasks, where participants produced prolonged vowel sounds for 3–5 s or for maximum duration, were commonly used to assess voice quality parameters [29, 30, 32, 33, 35, 37–39, 41–44]. Structured speech tasks included counting sequences [32, 33, 38, 39, 41–45]. Spontaneous speech samples were collected through structured interviews [32–35, 38–45] and picture descriptions [31–36, 38, 40, 42, 44]. Some studies also incorporated cognitive–linguistic tasks such as problem‐solving interactions and backward spelling to evaluate speech under cognitive load [35, 38, 39].

**Table 3:** Summary of the speech tasks used in the included publications.

Reference(s)	Automatic speech	Cognitive tasks	Connected speech	Family discussions	Free speech	Structure interviews	Picture description	Word production	Digit tasks	Text reading	Counting	Sustained vowels	Pitch glide tasks
Albuquerque et al. [35]	X	X	—	—	X	X	X	X	—	X	—	X	—
Alpert et al. [39]	X	X	—	—	X	X	—	—	—	X	X	X	—
Berardi et al.[40]	—	—	—	—	—	X	X	—	X	X	—	—	—
Cannizzaro et al. [45]	—	—	—	—	X	X	—	—	—	X	X	—	—
Cummins et al. [34]	—	—	—	—	—	X	X	—	X	X	—	—	—
Ellgring and Scherer [43]	—	—	—	—	X	X	—	—	—	X	X	X	X
Flint et al. [32]	—	—	—	—	X	X	X	X	—	X	X	X	X
Kim et al. [36]	—	—	—	—	—	—	X	—	X	X	—	—	—
Lu et al. [30]	—	—	X	—	—	—	—	—	—	X	—	X	—
Mundt et al.[44]	X	—	—	—	X	X	X	X	—	X	X	X	—
Mundt et al.[42]	X	—	—	—	X	X	X	X	—	X	X	X	—
Nilsonne [33]	—	—	—	—	X	X	X	—	—	X	X	X	X
Ooi et al. [37]	—	—	X	X	—	—	—	—	—	X	—	X	—
Seifpanahi et al. [41]	—	—	—	—	X	X	—	—	—	X	X	X	—
Talavera et al.[38]	—	X	X	—	X	X	X	X	—	X	X	X	—
Songur et al. [29]	—	—	X	—	—	—	—	—	—	X	—	X	—
Wang et al.[31]	—	—	—	X	—	—	X	—	X	X	—	—	—

Recording methodology varied considerably across studies. Earlier studies typically employed analog systems in sound‐treated environments, while recent research has adopted digital recording technology. Microphone placement was generally standardized at distances ranging from 6 inches (around 15 cm) [29, 32] to 12 inches (around 30 cm) [36] from the speaker’s mouth, with some studies using head‐worn condenser microphones [39] or neck‐mounted accelerometers [33] for consistent signal acquisition. Sampling rates ranged from 8 kHz (particularly in telephone‐based recordings) [42, 44] to 44.1 kHz [29, 35] with bit depths of 8–16 bits [31, 45]. Signal preprocessing typically included noise reduction [36], filtering [33, 38], and segmentation of voiced versus unvoiced speech components [31, 37, 40]. Several studies standardized recording times to control for diurnal mood variations, while more recent research has explored remote data collection through telephone systems and smartphone microphones.

### 3.2. Meta‐Analysis of the Association Between fo and Depression

**Figure 5:**
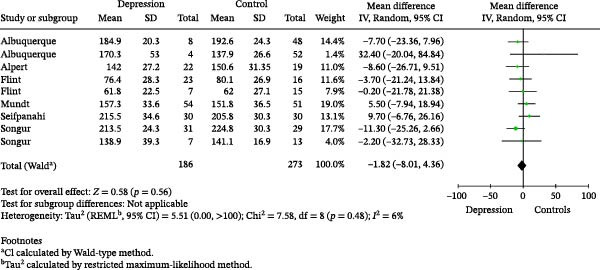
Forest plot and statistics of the random meta‐analysis of the relationship between fundamental frequency and depression.

We identified several voice acoustic parameters reported in the literature investigating depression. These parameters included fo, standard deviation of the fo (fo SD), jitter, shimmer, sound pressure levels (SPLs), standard deviation of the SPL (SPL SD), NHR, HNR, CPP smoothed (CPPS), and MPT. Across the 17 included publications, only six reported fo for cases and controls; therefore, the meta‐analysis was performed exclusively for fo and depression. Figure 5 shows the forest plot of the meta‐analysis on the association between fo and depression. Overall, the MD suggests a decreased fo of 1.82 Hz among participants identified with depression compared to participants identified as the control group. However, the difference between the groups was not statistically significant (*Z* test = 0.58; *p*‐value 0.56).

### 3.3. Publication Bias

**Figure 6:**
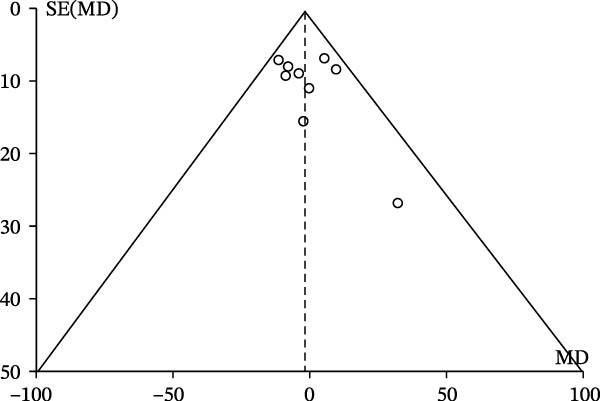
Funnel plot of the random meta‐analysis of the relationship between fundamental frequency and depression.

The funnel plot illustrates the relationship between the MD and the standard error of the MD (SE(MD)) across multiple studies reporting fo in participants with and without depression. Most data points cluster near the top center of the plot, indicating smaller standard errors and MDs close to zero, which suggests consistency in the results of these studies. However, the presence of an outlier further from this central cluster on both axes suggests some degree of heterogeneity (Figure 6).

### 3.4. Quality Assessment of the Included Publications

After assessing all publications using the Quality Assessment Tool for Quantitative Studies developed by the Effective Public Health Practice Project (EPHPP), eight studies were rated as methodologically “weak” [30, 31, 36, 38–41, 45], eight as “moderate” [29, 32–35, 42–44], and one as “strong” [37] (Table 4). Studies classified as weak most commonly demonstrated limitations related to study design and control of confounding variables, as well as insufficient reporting of participant selection procedures and outcome assessor blinding. In several cases, small or convenience samples, as well as cross‐sectional designs, further reduced methodological rigor.

**Table 4:** Quality assessment scores of the included publications.

Title	Selection bias	Study design	Confounders	Blinding	Data collection	Dropouts	Global rating
Determination of the voice parameters in adult individuals with rheumatoid arthritis and the relation of voice with depression and inflammation	Moderate	Moderate	Strong	Weak	Moderate	Moderate	Moderate
Exploring the characteristics of functional dysphonia by multimodal methods	Moderate	Weak	Strong	Weak	Strong	Moderate	Weak
Fast and accurate assessment of depression based on voice acoustic features: a cross‐sectional and longitudinal study	Moderate	Moderate	Strong	Weak	Strong	Weak	Weak
Acoustic analysis in the differentiation of Parkinson’s disease and major depression	Moderate	Weak	Strong	Moderate	Strong	Moderate	Moderate
Acoustic analysis of speech variables during depression and after improvement	Moderate	Moderate	Strong	Weak	Strong	Strong	Moderate
Analysis of acoustic space variability in speech affected by depression	Moderate	Strong	Strong	Weak	Strong	Moderate	Moderate
Association between acoustic speech features and non‐severe levels of anxiety and depression symptoms across lifespan	Moderate	Weak	Strong	Moderate	Strong	Moderate	Moderate
Automatic depression detection using smartphone‐based text‐dependent speech signals: deep convolutional neural network approach	Moderate	Weak	Strong	Weak	Strong	Moderate	Weak
Prediction of major depression in adolescents using an optimized multi‐channel weighted speech classification system	Moderate	Moderate	Strong	Moderate	Strong	Strong	Strong
Quantitative measurement of depression through speech analysis	Moderate	Weak	Strong	Weak	Strong	Moderate	Weak
Reflections of depression in acoustic measures of the patient’s speech	Moderate	Weak	Strong	Weak	Strong	Moderate	Weak
Relative importance of speech and voice features in the classification of schizophrenia and depression	Moderate	Weak	Strong	Weak	Strong	Weak	Weak
The Association between Depression Severity, Prosody, and Voice Acoustic Features in Women with Depression	Moderate	Weak	Strong	Weak	Strong	Moderate	Weak
Vocal acoustic biomarkers of depression severity and treatment response	Moderate	Moderate	Strong	Weak	Strong	Moderate	Moderate
Vocal indicators of mood change in depression	Moderate	Moderate	Strong	Weak	Strong	Strong	Moderate
Voice acoustic measures of depression severity and treatment response collected via interactive voice response (IVR) technology	Moderate	Moderate	Strong	Weak	Strong	Strong	Moderate
Voice acoustical measurement of the severity of major depression	Moderate	Weak	Strong	Weak	Strong	Weak	Weak

Studies rated as moderate generally met the criteria for appropriate data collection methods and outcome measurement; however, they often lacked a comprehensive description of blinding. Only one study achieved a strong methodological rating, reflecting robust study design, clear reporting of recruitment procedures, validated outcome measures, and appropriate control of confounding factors.

Overall, the predominance of studies rated as weak or moderate suggests that the current evidence base should be interpreted with caution.

## 4. Discussion

This bibliometric analysis, systematic literature review, and meta‐analysis were conducted to consolidate and evaluate the current evidence regarding the relationship between voice acoustic parameters and depression. We identified several voice acoustic parameters reported in the literature investigating depression. These parameters included fo, fo SD, jitter, shimmer, SPLs, SPL SD, NHR, HNR, CPPS, and MPT. Interestingly, across the 17 included publications, only six reported fo and fo SD; two papers reported jitter, shimmer, or HNR; one paper reported SPL, SPL SD, NHR, CPPS, or MPT.

The presence of these acoustic parameters across multiple studies indicates that they may be characteristic features of depressive speech patterns. While fo, jitter, and shimmer have traditionally provided valuable initial insights into depression‐related voice changes, research has begun to shift toward more advanced acoustic features, including MFCCs, HNR, and CPP. These measures offer a more comprehensive characterization of voice quality, vocal tract function, and articulatory precision. Additionally, machine learning models leveraging these features have shown promising accuracy in distinguishing depressed and nondepressed speech.

This substantial heterogeneity in the acoustic metrics reported across studies limited the extent to which findings could be meaningfully compared or synthesized. As noted, only fo was reported with sufficient numerical detail to allow quantitative pooling. This restricted evidence base directly affects the interpretability of the meta‐analytic results. Moreover, although the pooled analysis identified a decrease of 1.82 Hz in fo among participants with depression, the difference was not statistically significant.

Regarding depression, our systematic review showed considerable heterogeneity in the methodological approaches used to assess depression across the included studies. The HAM‐D emerged as the predominant assessment tool, utilized in 11 of the 17 studies. This result aligns with findings from previous meta‐analyses, such as the work by Cummins et al. [9], which identified HAM‐D as the gold standard clinician‐administered scale in voice‐based depression research. The widespread adoption of HAM‐D likely stems from its robust psychometric properties and established validity in clinical settings [46]. Other trends were also identified. The BDI appeared frequently, particularly valued for its self‐report format that reduces clinician burden. The PHQ‐9 was often implemented alongside HAM‐D, while the QIDS was utilized as a complementary measure in multiple studies. This diverse approach to depression assessment reflects the multifaceted nature of the condition and acknowledges that no single instrument captures all relevant dimensions of depressive symptomatology [47].

Notably, our findings indicate a methodological evolution toward integrated assessment approaches. Recent studies employed multiple assessment instruments simultaneously, combining standardized diagnostic criteria (DSM‐IV/DSM‐5) with validated rating scales (HAM‐D and PHQ‐9) and clinical evaluations [36, 40]. This triangulation approach addresses limitations inherent in any single assessment methodology and provides a more comprehensive clinical picture.

The temporal distribution of assessment methodologies revealed a progression from reliance on a single scale in earlier studies [32, 33] to more sophisticated, multimethod approaches in contemporary research. This methodological refinement parallels advancements in our understanding of depression as a heterogeneous condition with varied symptom profiles and biological underpinnings. Contemporary depression research increasingly recognizes that different symptom clusters (e.g., psychomotor, cognitive, and emotional) may manifest distinct biological correlates [40]. In the context of voice acoustic analysis, this limitation is particularly relevant, as certain voice parameters may correlate specifically with psychomotor symptoms, while others may relate more closely to emotional or cognitive dimensions [40].

Our review identified several studies comparing acoustic patterns in depression to those observed in other conditions. This comparative approach provides valuable context for determining whether certain voice parameters are depression‐specific or reflect more general psychopathological processes. Studies have examined acoustic similarities and differences between depression and conditions such as schizophrenia, Parkinson’s disease (PD), and posttraumatic stress disorder (PTSD). Acoustic similarities between individuals with depression and those with PD have been documented, including decreased second formant location and improper glottal closure. These shared features may reflect psychomotor retardation present in both conditions despite their distinct etiologies [32]. In studies differentiating depression from schizophrenia, voice pathology features emerged as particularly important diagnostic markers due to their correlation to the positive symptoms of schizophrenia, such as formal thought disorder [40]. Given the growing body of evidence supporting the role of speech and voice acoustic parameters as biomarkers for depression, introduced in the present work, machine learning techniques have emerged as a promising approach to enhance diagnostic accuracy. Traditional models have relied on handcrafted acoustic features, employing classifiers such as support vector machines and random forests. More recently, deep learning architectures, including convolutional neural networks and long short‐term memory networks, have been explored, leveraging depression rating scales as the ground truth for model training and validation [21, 22]. These advanced techniques demonstrate significant potential in speech depression detection, with recent reviews reporting pooled accuracy estimates of 0.87 based on eight studies [21]. This reinforces the viability of speech‐based models as an initial screening tool, helping clinicians identify individuals at risk and prompting them to seek further mental health evaluation.

Despite the promising performance of these models, they must be used cautiously and as complementary tools rather than standalone diagnostic solutions. One key limitation is the generalizability of these models. Many have been trained on relatively small, domain‐specific datasets, making them prone to overfitting. As a result, their performance can degrade when applied to speech samples from different languages, dialects, recording environments, or recording setups. These factors significantly influence acoustic features and, consequently, model outcomes [48, 49]. Additionally, model explainability remains a major concern, particularly for deep learning‐based approaches, where understanding the decision‐making process and interpreting model outputs remains challenging. To fully harness the potential of these technologies, ongoing efforts should focus on finding a balance between performance and interpretability, developing more robust and generalizable models, and building larger, more diverse datasets. By addressing these challenges, machine learning models can play a crucial role in the future of depression screening, enabling early identification and broader accessibility to mental health services.

Lastly, the clinical implications of our findings are considerable, particularly in addressing the limitations of traditional depression assessment methods that rely on subjective self‐report and clinical observation. By integrating voice acoustic measures into digital health platforms, clinicians may obtain real‐time insights into patients’ mental health status, potentially reducing barriers to care for individuals in remote or underserved populations.

### 4.1. Limitations and Future Opportunities

As with all studies, there are limitations to this work and opportunities for future research. One key limitation is the variability in methodologies, including differences in depression assessment tools, voice recording conditions, and acoustic analysis techniques. This heterogeneity hampers cross‐study comparisons and meta‐analytic integration. Additionally, this review found a predominance of cross‐sectional designs, with only two studies incorporating longitudinal assessments. This temporal limitation restricts our understanding of how voice parameters fluctuate with depression trajectories across time. Another limitation is the quality assessment of the studies, which showed that only one out of 17 studies achieved a “strong” methodological rating, with eight classified as “weak” and eight as “moderate.” This distribution indicates substantial room for methodological improvement. Fourth, the current literature is a limited exploration of mechanisms linking voice acoustic parameters to the neurobiological underpinnings of depression. Our review identified no studies that directly investigated relationships between voice acoustics and neuroimaging or neurophysiological measures in depression. Fifth, while the included studies examined a wide range of acoustic parameters, most parameters were reported in only a small number of studies, limiting opportunities for quantitative synthesis. Consequently, only fo met the criteria for meta‐analysis. Standardized reporting of acoustic data is needed to enable more comprehensive comparisons in future research. Six, a key limitation of the meta‐analysis, relates to the potential influence of gender on fo. Although sex‐stratified analyses would have strengthened interpretability and reduced the risk of confounding, only three of the six included studies reported fo data separately by sex. The remaining studies provided aggregated values, which precluded a consistent sex‐specific pooled analysis. Given the small number of eligible studies and incomplete stratified reporting, conducting subgroup analyses would have resulted in limited statistical power and potentially unstable estimates. Consequently, residual confounding by gender cannot be excluded, and findings related to fo differences should be interpreted with appropriate caution. Finally, most studies in our review employed controlled, laboratory‐based voice recording protocols, which may not capture the naturalistic speech patterns encountered in real‐world settings. The ecological validity of findings from such controlled environments remains uncertain.

Future research may prioritize several methodological advancements to strengthen voice‐based depression assessment. Standardization of recording protocols, establishment of normative acoustic benchmarks, and validation against established diagnostic criteria (DSM‐5 and ICD‐11) and severity measures (HAM‐D and PHQ‐9) are essential. Longitudinal designs are needed to track how acoustic markers evolve throughout depression and treatment, while cross‐cultural validation studies and linguistically diverse samples will ensure global applicability and determine whether acoustic features require language‐specific calibration. Moreover, studies should address identified methodological weaknesses, particularly blinding procedures and selection bias, while adopting multimodal approaches that integrate voice analysis with neurobiological assessments. Enhanced collaboration with linguistic experts will strengthen the understanding of language‐specific features and facilitate cross‐linguistic comparisons, ultimately expanding datasets to improve generalizability across diverse populations and contexts.

Our analysis suggests several promising interdisciplinary collaborations that could accelerate progress in this field. The partnership between psychology, psychiatry, and speech sciences represents a fundamental collaboration, combining clinical expertise in depression phenomenology with specialized knowledge of voice production and analysis. Furthermore, the integration of computational sciences with clinical psychology offers another powerful interdisciplinary approach. As demonstrated by Kim et al. [36], the application of advanced machine learning techniques to voice analysis requires collaboration between data scientists, computer engineers, and mental health professionals. This computational psychology approach leverages technological expertise to address clinical challenges while ensuring that computational models remain grounded in clinical reality.

Collaborative efforts can also help develop standardized protocols and ensure that voice‐based depression assessment tools are both accurate and accessible across diverse populations. By addressing these challenges, future research can move closer to establishing voice analysis as a reliable tool for mental health assessment.

Neuroscience represents another critical discipline for future collaboration. Understanding the neurobiological mechanisms underlying depression‐related voice changes requires the integration of voice analysis with neuroimaging and neurophysiological approaches. Our review also identified linguistics as a prominently represented discipline, reflecting the importance of language expertise in voice analysis. Future collaborations should strengthen the integration of linguistic knowledge, particularly regarding language‐specific features and cross‐linguistic comparisons.

## 5. Conclusion

In conclusion, our results suggest the potential of voice acoustic parameters as noninvasive, cost‐effective biomarkers for measuring and monitoring depression symptomatology. Although the meta‐analysis suggests a nonsignificant difference in average values of fo between subjects with and without depression, the trend of decreased fo of 1.82 Hz among participants identified with depression compared to participants identified as the control group indicates that fo offers a promising avenue for early detection and continuous monitoring of depressive symptoms, particularly in remote or underserved populations. Future research should focus on developing more robust and generalizable models, expanding datasets, and integrating voice analysis with established clinical assessment tools to enhance the accuracy and accessibility of depression diagnosis and treatment.

## Funding

This study was funded by the National Institute on Deafness and Other Communication Disorders (Grant R01DC012315).

## Disclosure

After using ChatGPT‐4o (OpenAI), the authors reviewed and edited the content as needed and took full responsibility for the content of the publication. The content is solely the responsibility of the authors and does not necessarily represent the official views of the National Institutes of Health.

## Conflicts of Interest

The authors declare no conflicts of interest.

## Data Availability

The data that support the findings of this study are available from the corresponding author upon reasonable request.
